# Molecular and nanoscale evaluation of N-cadherin expression in invasive bladder cancer cells under control conditions or GW501516 exposure

**DOI:** 10.1007/s11010-020-03771-1

**Published:** 2020-06-09

**Authors:** Céline Elie-Caille, Isabelle Lascombe, Adeline Péchery, Hugues Bittard, Sylvie Fauconnet

**Affiliations:** 1 FEMTO-ST Institute, Univ. Bourgogne Franche-Comté, CNRS, ENSMM, UTBM, Besançon, France; 2 Univ. Bourgogne Franche-Comté, EA3181, LabEx LipSTIC ANR-11-LABX-0021, 25030 Besançon, France; 3 Service Urologie et Andrologie, CHU Besançon, 25000 Besançon, France

**Keywords:** Bladder cancer, Cell adhesion, Cadherin, GW501516, PPARβ/δ, Migration

## Abstract

N-cadherin is a transmembrane glycoprotein expressed by mesenchymal origin cells and is located at the adherens junctions. It regulates also cell motility and contributes to cell signaling. In previous studies, we identified that its anomalous expression in bladder carcinoma was a tumor progression marker. A pharmacological approach to inhibit N-cadherin expression or to block its function could be relevant to prevent disease progression and metastasis development. The morphological exploration of T24 invasive bladder cancer cells by atomic force microscopy (AFM) revealed a spindle-like shape with fibrous structures. By engaging force spectroscopy with AFM tip functionalized with anti-E or anti-N-cadherin antibodies, results showed that T24 cells expressed only N-cadherin as also demonstrated by Western blotting and confocal microscopy. For the first time, we demonstrated by RTqPCR and Western blotting analyses that the peroxisome proliferator-activated receptor β/δ (PPARβ/δ) agonist GW501516 significantly decreased N-cadherin expression in T24 cells. Moreover, high non-cytotoxic doses of GW501516 inhibited confluent T24 cell wound healing closure. By using AFM, a more sensitive nanoanalytical method, we showed that the treatment modified the cellular morphology and diminished N-cadherin cell surface coverage through the decreasing of these adhesion molecule-mediated interaction forces. We observed a greater decrease of N-cadherin upon GW501516 exposure with AFM than that detected with molecular biology techniques. AFM was a complementary tool to biochemical techniques to perform measurements on living cells at the nanometer resolution level. Taken together, our data suggest that GW501516 could be an interesting therapeutic strategy to avoid bladder cancer cell spreading through N-cadherin decrease.

## Introduction

Urothelial carcinoma of the bladder is a significant cause of morbidity and mortality with almost 550,000 new cases and 200,000 deaths in 2018 worldwide [1]. At diagnosis, 75% of cases are non-muscle-invasive bladder cancers (NMIBC) with a risk of progression according to grade and stage [2]; the remaining 25% invade the detrusor muscle (MIBC) upon presentation with a poorer prognosis. Patients with NMIBC are treated with transurethral resection associated or not with intravesical therapy [3]. Standard care of organ-confined MIBC includes radical cystectomy with or without neoadjuvant chemotherapy or chemoradiotherapy [4]. However, even after treatment, 50% of patients will relapse with metastasis development within 2 years and will die of their disease [5]. Reliable biomarkers of progression are needed to improve the biologic assessment of bladder cancers for a better disease management. In a previous work, whereas the normal urothelium does not express N-cadherin (Neuronal-cadherin), we showed that the expression of this adhesion molecule appeared in T1-stage NMIBC confined to the lamina propria, invading the connective tissue surrounding the urothelium. Thus, N-cadherin acted in an invasive mode in bladder carcinoma and was an independent prognostic biomarker of pT1 tumor progression [6].

N-cadherin as well as E-cadherin (Epithelial-cadherin) belongs to the classical cadherin family. They are transmembrane glycoproteins involved in adherens junction formation, crucial for the maintenance of tissue organization during development and in adult organisms. They consist of an ectodomain composed of five repetitive extracellular domains (EC1 to EC5) possessing binding sites for calcium, a single transmembrane domain, and a C-terminal cytoplasmic region directly associated with β-catenin or γ-catenin which interacts with actin cytoskeleton through α-catenin [7]. Apart from their function in intercellular adhesion, cadherins are key regulators of cell motility and contribute to cell signaling [8]. Alterations in the expression and function of cadherins play a key role in cancer progression. E-cadherin suppression in epithelial cells is associated with de novo expression of mesenchymal markers such as N-cadherin. This cadherin switching has been reported in different cancers [9] and is a hallmark of the Epithelial-Mesenchymal Transition (EMT). EMT is characterized by the remodeling of epithelial cell architecture including loss of cell–cell adhesion, loss of cell polarity, cytoskeletal reorganization, morphological changes, and acquisition of migratory and invasive properties. It is a crucial factor for tumor cell invasion and metastasis development [10]. A meta-analysis showed that the upregulation of N-cadherin was associated with more aggressive carcinoma, linked to higher tumor grade and stage, as well as lymph node metastases [11]. In this context, a pharmacological approach to block N-cadherin function or to decrease its expression in cancer cells could be relevant to prevent primary tumor progression and control dissemination of metastases.

In the present work, we assessed whether GW501516 could inhibit N-cadherin expression of invasive bladder cancer cells and impact their migratory capacities. GW501516 is a high-affinity chemically synthesized activator of the Peroxisome Proliferator-Activated Receptor β/δ (PPARβ/δ). PPARβ/δ is a ligand-inducible transcription factor belonging to the nuclear receptor superfamily. Its heterodimerization with RXR (retinoid × receptor) is required to bind to a PPRE (peroxisome proliferator response element) *cis*-regulatory sequence located in the promoter region of target genes [12]. The role of PPARβ/δ in cancer is complex and controversial. Few studies reported that GW501516 could negatively modulate cell migration and invasion of cancer cells. Indeed, GW501516 decreased MMP-9 (Matrix Metalloproteinase-9) expression in pancreatic cancer cells and, consequently, their invasion capacity [13]. It blocked both cell migration and invasion of breast cancer cells [14]. It inhibited tumor growth in mice xenografted with MDA-MB-231 cells through PPARβ/δ/c-MYC interaction [15]. It induced nasopharyngeal carcinoma C666-1 cell apoptosis [16] and decreased the proliferative capacity of hepatoma cells [17]. In a previous study, we reported that a high concentration of GW501516 (25 µM) induced cytotoxic effects in proliferating T24 invasive bladder cancer cells, including a decrease in cell viability, a G2/M cell cycle arrest, and the triggering of cell death through both extrinsic and intrinsic apoptotic pathways via Bid cleavage [18]. In particular, the drug led to an increase of the Bax/Bcl-2 ratio, a mitochondrial dysfunction associated with the dissipation of ∆Ψm, and the release of the cytochrome c from the mitochondria to the cytosol.

In this work, we aimed at exploring the expression level of N-cadherin in invasive bladder cancer cells upon GW501516 exposure by both molecular biology techniques such as RTqPCR and Western blotting and atomic force microscopy (AFM) using a tip functionalized with a monoclonal antibody directed against this adhesion molecule. AFM is a mighty nanoanalytical tool for studying biological samples under liquid, in pathological or physiological conditions, and at the scale of a single cell. It allows to characterize cells and their modification upon drug exposure or function alteration, in terms of cell surface topography or cell adhesion [19]. For the first time, we demonstrated that the PPARβ/δ activator from a concentration of 15 µM decreased the full length N-cadherin at the mRNA and protein level and significantly reduced its cell surface coverage through the measurements of the interaction forces involving this adhesion molecule.

## Materials and methods

### Chemicals

The PPARβ/δ activator GW501516 was provided by Enzo Life Sciences (Villeurbanne, France). Ciglitazone (PPARγ agonist) was purchased from Cayman Chemical (Ann Arbor, MI). The final concentration of organic solvent (dimethylsulfoxide) was less than 0.1% which had no effect on cell viability.

### Cell lines and culture

The human bladder cancer cell lines RT4 and T24 were obtained from ATCC. Cells were maintained in Mc COY’s 5a medium supplemented with 10% fetal calf serum (FCS) (Lonza, Basel, Switzerland), 1% antibiotic antimycotic mixture (100 µg/mL streptomycin, 100 U/mL penicillin), 2 mM glutamine (Sigma-Aldrich, Saint Quentin Fallavier, France) at 37 °C in a humidified 5% CO_2_, 95% O_2_ air incubator. They were tested for the absence of mycoplasma before the beginning of experiments.

### Atomic force microscopy

Cells were seeded on coverslides in 4-well plates at a density of 200,000 RT4 cells/well or 80,000 T24 cells /well in 5% FCS Mc COY’s 5a medium for 24 h. After two washes, they were stimulated in FCS-free medium with or without 15 µM GW501516 for 24 h.

AFM imaging was carried out on a Nanowizard III AFM (JPK Instruments, Germany). The contact mode was used on fixed cells (with an applied force of 0.1 V), the QI mode was used for alive cell imaging [20–22], all in liquid. Force spectroscopy in force mapping was used for cadherin/anti-cadherin antibody measurement interactions and cadherin mapping on cells. For that, the coverslide was installed in the biocell, in 1 mL PBS 1X, at a regulated temperature of 37 °C. V-shaped silicon nitride cantilevers (PNP-TR-50, from Nano and more) with a spring constant of 0.08 N/m were used. For force mapping, cell maps of 16 × 16 or 32 × 32 pixels were realized, with the following parameters: setpoint of 0.3 V and, after the cantilever calibration, sensitivity and spring constant of 29.03 nm/V and 0.019 N/m, respectively. AFM probes, made of silicon nitride, were functionalized by 1% APTES (3-(Aminopropyl)triethoxysilane) in toluene during 2 h, washed extensively with toluene, and then with ethanol. The second step consisted in an incubation in 0.2% glutaraldehyde solution during 10 min, followed by extensive washing with water. A naked tip was used as a negative control. The modified tips were then incubated in 50 µg/mL primary antibody solution (N-cadherin GC-4 clone directed against the extracellular domain, N-cadherin 3B9 clone directed against the intracellular domain, E-cadherin HECD-1 clone directed against the extracellular domain) during 30 min, then washed with PBS 1X. Finally, the functionalized tip was saturated by incubation in 2 mg/mL RSA (rat serum albumin) solution during 30 min (as described in [23]).

### Immunocytofluorescence and confocal microscopy

T24 cells grown on coverslides in 6-well plates at a density of 150,000 cells/well in Mc COY’s 5a medium containing 5% FCS were washed twice with cold PBS 1X and fixed for 10 min in 4% paraformaldehyde (Euromedex, Souffelweyersheim, France) at room temperature (RT). After three washes, cells were incubated for 10 min in 50 mM NH_4_Cl. Cells were washed three times in PBS 1X and permeabilized or not for 4 min in 0.1%Triton X-100 (Sigma). After a 5 min wash in PBS 1X, cells were saturated for 30 min at RT in PBS 1X containing 1% BSA and 1% FCS. Cells were then incubated for 1 h at RT with an anti-N-cadherin antibody (1:50 for 3B9 clone; 1:100 for GC-4 clone). After three washes in PBS 1X, cells were incubated with an FITC-conjugated anti-mouse IgG secondary antibody (1:1000) (Sigma). Coverslides were washed and mounted on slides with VECTASHIELD® mounting medium and were observed by fluorescent microscopy using the Olympus FluoView 1000 (Olympus).

### DAPI nuclear staining

T24 cells were seeded on coverslides in 6-well plates at a density of 150,000 cells/well in Mc COY’s 5a medium containing 5% FCS. After 24 h, they were washed once with PBS 1X and were fixed in 4% paraformaldehyde for 10 min at RT. Fixed cells were then washed three times with PBS 1X and permeabilized by immersion in 0.1% Triton X-100 for 10 min. After three further washes with PBS 1X, cells were incubated for 30 s at RT in 1.5 µg/mL DAPI labeling solution (diluted at 1/1000 in PBS 1X) (AAT Bioquest Inc., Sunnyvale, CA, USA). DAPI solution was aspirated and the cells were washed three times in PBS 1X. The coverslides were mounted on slides with VECTASHIELD® mounting medium and observed by fluorescent microscopy using the Olympus FluoView 1000 (Olympus).

### Protein extraction and Western blotting analysis

After treatment or not (control cells) with GW501516, cells were washed with cold PBS 1X and scraped in lysis buffer RIPA (50 mM Tris–HCl pH 7.4, 150 mM NaCl, 1 mM EDTA, 1% Nonidet P-40, 0.5% sodium desoxycholate) supplemented with protease inhibitors (Roche Diagnostics, Meylan, France). Then, whole cell lysates were sonicated and centrifuged at 10,000×*g* for 10 min at 4 °C. Protein concentration was estimated using the Bradford protein assay according to the manufacturer’s recommendations (Bio-Rad, Marnes-la-Coquette, France). Total protein extracts (30 µg) were solved in Laemmli buffer (Bio-Rad) and separated by a 12% SDS-PAGE. Proteins were transferred onto PVDF membranes (GE Healthcare, England) and non-specific binding was blocked in TBS-Tween 20 buffer (0.5 mM Tris–HCl, 45 mM NaCl, 0.05% Tween 20, pH 7.4) containing 5% non-fat milk. Membranes were incubated with the following appropriate primary antibodies: anti-β-actin (clone AC-15, 1:8000) and anti-N-cadherin (clone GC-4, 1:1000) were from Sigma. Anti-N-cadherin (clone 3B9, 1/2000) and anti-E-cadherin (clone HECD-1, 1:1000) were from Fisher Scientific (Illkirch, France). Anti-cleaved caspase 3 (#9661, 1:1000) was from Cell Signaling (Ozyme, St Quentin en Yvelines, France). Anti-PARP (clone 4C10-5, 1:1000) was obtained from BD Pharmingen (BD Biosciences, Le Pont de Claix, France). Bound primary antibodies were detected using HRP-conjugated secondary antibodies: anti-rabbit IgG (1:5000) or anti-mouse IgG (1:5000 or 1:10,000) provided from BD Pharmingen. Proteins were visualized by using enhanced chemiluminescence detection method (GE Healthcare) followed by film exposure (Hyperfilm ECL, GE Healthcare) or by using ChemiDoc XRS+ with image lab software (Bio-Rad). Densitometric analysis was performed both with the software Image J and ChemiDoc XRS+ with image lab software.

### RNA isolation, cDNA synthesis, and quantitative real-time PCR analysis

Total RNA were extracted using TRI reagent (Euromedex). A RNase-free DNase I treatment was carried out for removing contaminating genomic DNA (Fisher Scientific) according to the manufacturer's instructions. Complementary DNA synthesis was performed from total RNA with 200 U MMLV Reverse Transcriptase (Fisher Scientific) and 500 ng oligo(dT) primers (Fisher Scientific) following the manufacturer’s guidelines. PCR assays were performed with the 7500 Real Time PCR System (Applied Biosystems, Saint-Aubin, France) using TaqMan technology in a final volume of 25 μL containing 12.5 μL of TaqMan Gene Expression PCR Master Mix (Applied Biosystems), 5 µL of cDNA diluted 1:20, 100 nM of *cdh2*, or 300 nM of *atp5β* TaqMan probe (Eurogentec, Seraing, Belgium), and 1 µM of each primer (Eurogentec) for *cdh2* or 500 nM for *atp5β*. Specific primers and probe were the following for *cdh2*: forward 5′- tgggaatccgacgaatgg -3′; reverse 5′- gcagatcggaccggatactg -3′; probe: 6-FAM-tgaaagacccatccacgctgagcc-BHQ-1, and for the housekeeping gene *atp5β* is used as a reference gene for normalization: forward 5′- tactgtcgcgtgccattgct -3′; reverse 5′- cacgggcaacatcgtaatgc -3′; probe: 6-FAM-atcccaacattgttggcagt-BHQ-1. The Taq polymerase was activated at 50 °C for 2 min, followed by a denaturation step at 95 °C for 10 min. Then, PCR mixtures were subjected to 40 cycles of amplification. The following PCR cycle settings were used: denaturation at 95 °C for 15 s and hybridization/elongation at 60 °C for 1 min. Each reaction was run in triplicate with three independent triplicates. We calculated the relative mRNA expression level using a comparative C_T_ method [24]. The raw threshold cycle (C_T_) value was normalized to the housekeeping gene for each sample to obtain the ΔC_T_. The normalized ΔC_T_ value was calibrated to the control cell samples to obtain the ΔΔ*C*_T_ value.

### RNA interference and cell transfection

Control siRNA (negative control for evaluating RNAi off-target effects, sc-37007) and *PPARβ/δ* (sc-36306)-specific siRNA (pool of 3 target-specific 19–25 nt siRNAs) were from Santa Cruz Biotechnology. T24 cells were seeded in 24-well plates (80,000 cells/well) and cultured in Mc COY’s 5a medium with 5% FCS, but without antibiotics. After 24 h, at 70–80% confluence, cells were transfected with 50 nM siRNA using Lipofectamine™ 2000 reagent (Invitrogen, ThermoFisher Scientific, Illkirch, France) according to the manufacturer’s instructions. After 24 h transfection, cells were incubated in serum-free medium without (control cells) or with 15 µM GW501516 for 24 h more and then were harvested for protein extraction and Western blotting analysis.

### Scratch wound healing assay

T24 cells were seeded in 6-well plates at 300,000 cells/well and cultured until reaching approximately 100% confluence. A 100 μL pipette tip was used to create a vertical linear scratch in cell monolayers. The detached cells were removed by PBS 1X washing. Then, cells were incubated with fresh medium for 24 h in the absence or presence of 10% FCS or 15 µM GW501516. Images of cell migration were captured by an inverted light microscope (Olympus CKX41) (× 10 magnification) at 0 and 24 h after the injury. Cell migration was assessed by measuring gap size through using Image J software. Marks have been made on each well before seeding cells so that at each experimental time and in each condition, photos are taken at the same place. These marks are visible in black on the photos. The experiments were conducted in triplicate to obtain an average value.

### Cell death analysis by flow cytometry

T24 cells were seeded in triplicate (6000 cells/cm^2^) in 12-well plates and incubated in culture medium supplemented with 5% decomplemented FCS. After 24 h, they were exposed to 15 or 20 µM GW501516 for 24 h or to 40 µM ciglitazone (positive control for cell death) in serum-free culture medium. DNA fragmentation was measured by propidium iodide staining and fluorescence-activated cell sorting (FACS) analysis (FC 500 Beckman Coulter) as previously described [25]. Twenty thousand events were analyzed per sample and apoptosis was determined by the Sub-G1 DNA content with CXP software (Beckman Coulter).

### Data processing and statistical analysis

Quantitative imaging AFM mode enabled to register more than hundred force spectroscopy curves per condition. The curves registered on cells were overlayed in order to highlight a specific pattern and the interaction peak areas were measured. For Western blotting and RTqPCR experiments, data were expressed as mean ± SEM of three independent experiments performed in triplicate or as specified for each figure. The significant differences between groups were evaluated using the two-tailed unpaired Student’s *t* test. A *P* value < 0.05 was considered statistically significant.

## Results

### Bladder cancer cell morphology and topography

**Fig. 1:**
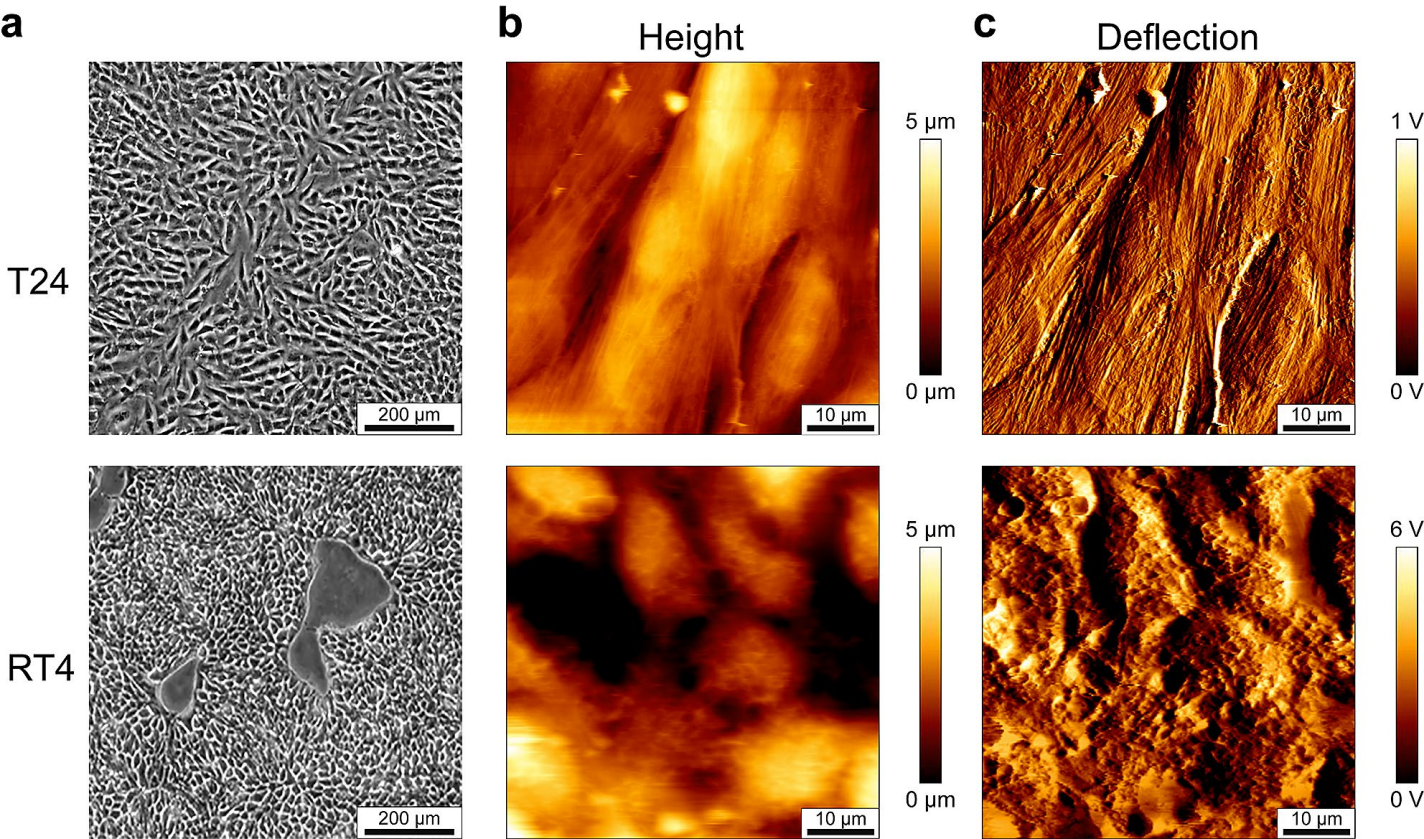
T24 and RT4 bladder cancer cell morphology and topography. **a** Images from control confluent cells by phase contrast microscopy. Scale bars: 200 µm. **b**, **c** AFM images obtained on control confluent cells, after glutaraldehyde fixation, in contact mode in liquid. **b** AFM height images. **c** AFM deflection images. Scale bars: 10 µm

Using AFM we carried out a morphological and topographical analysis on bladder cancer cells of different histologic grade. Cells were grown on glass slides, that were previously inserted in wells, and were characterized in the confluence state. T24 cells (derived from an undifferentiated grade 3 carcinoma; representative cells of a bad prognosis cancer) are elongated cells, with a length ranging from 23 to 30 µm, a width around 15 µm, and fibrous structures show up on the entirety of all cells, giving a cell roughness of 273 ± 84 nm. In comparison, RT4 cells (derived from a well-differentiated grade 1 papillary tumor; representative cells of a good prognosis cancer) are more round shape, with a mean diameter from 13 to 16.4 µm. Their surface present homogeneously distributed protrusions, but no fibrous structures, giving a roughness of 306 ± 114 nm (Fig. 1, Table 1).

**Table 1:** Characteristics of T24 and RT4 bladder cancer cells

Parameters	RT4	Untreated T24	GW501516-treated T24
Length (µm)	13.4 ± 3.0	26.4 ± 3.0	20.4 ± 5.0
Width (µm)	16.4 ± 4.0	16.5 ± 1.5	8.2 ± 3.1
Roughness Ra (nm)*	306 ± 114	273 ± 84	124 ± 16

*The roughness (Ra) value was determined on 10 × 10 µm area on cells. Ten cells were analyzed in each condition

### T24 cells express N-cadherin marker

**Fig. 2:**
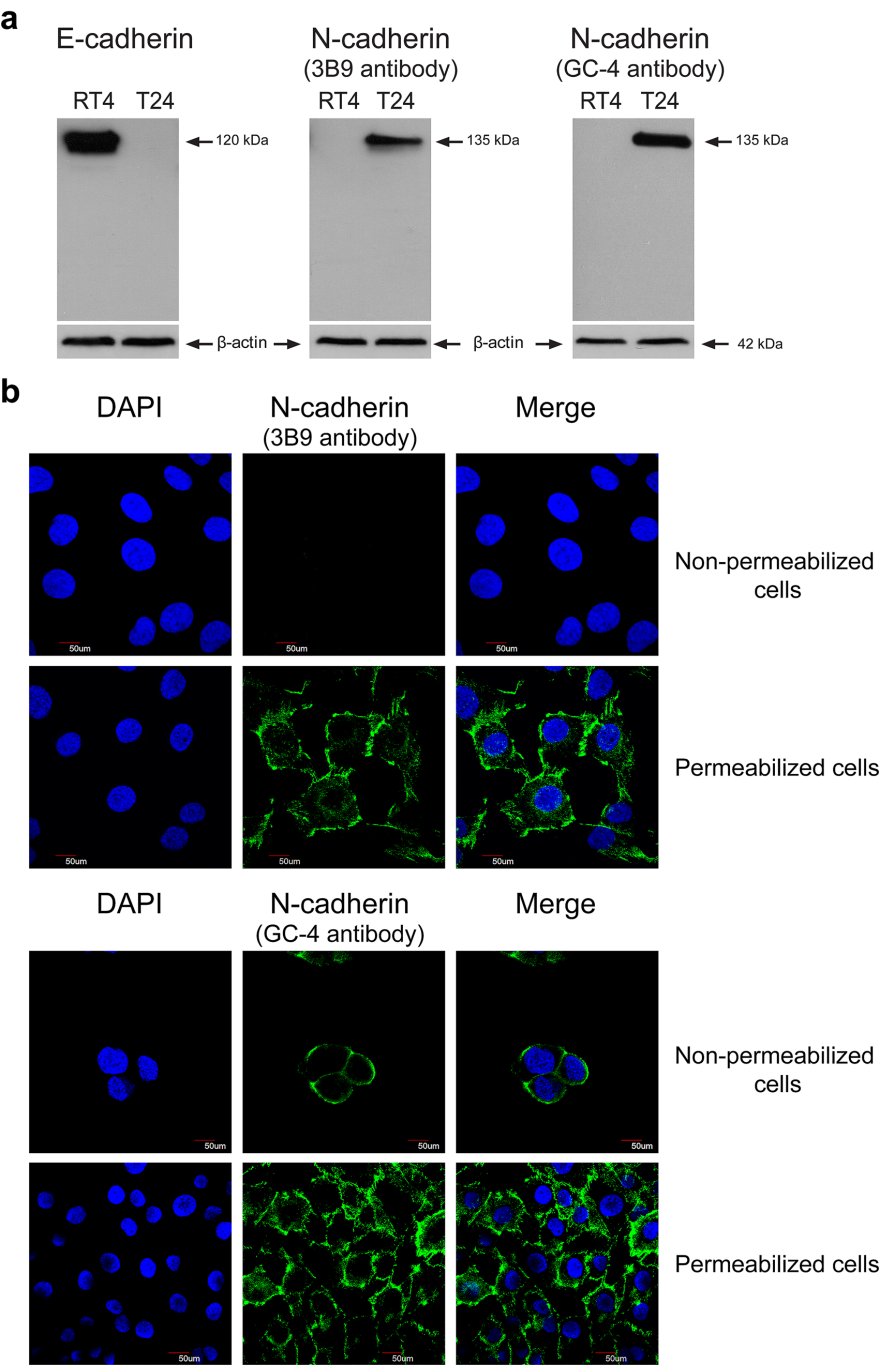
Validation of anti-cadherin antibody specificity. **a** Total protein extracts from RT4 and T24 cells were subjected to a Western blotting analysis for E-cadherin (clone HECD-1) and N-cadherin (clone 3B9 or GC-4) expression. β-actin was used as an internal loading control. **b** Representative confocal microphotographs of T24 cells immunostained for N-cadherin (green). Cells were permeabilized (× 60 magnification) or not (× 100 magnification) and N-cadherin detection was assessed by using the GC-4 antibody targeting the extracellular domain or the 3B9 antibody directed against the cytoplasmic part. Nuclei were stained in blue by DAPI. Scale bars: 50 µm. (Color figure online)

As we have already showed by a RT-PCR analysis, E-cadherin was expressed in RT4 cells, but not in T24 cells and N-cadherin was only expressed in T24 cells [6]. To characterize these cells for the expression of cadherins at the protein level and thus to validate the anti-cadherin antibodies used in our study, immunoblotting experiments were performed with total protein extracts from RT4 and T24 cells (Fig. 2a). Anti-E-cadherin antibody detected one E-cadherin band of 120 kDa size only in RT4 cells. Both anti-N-cadherin antibodies (the clone 3B9 directed against the cytoplasmic domain and the clone GC-4 directed against the extracellular domain) revealed the full lenght N-cadherin of 135 kDa only in T24 cells. The anti-cadherin antibodies reacted strongly with their targets and did not exhibit any aspecific hybridization or cross reactivity. Importantly, an image of the whole electrophoresis gel was shown and for each antibody a single protein band was detected. The results of immunoblots were confirmed by immunofluorescence staining carried out on T24 cells permeabilized or not by Triton X-100 (Fig. 2b). As expected, the membrane localization of N-cadherin was visualized in the presence of the 3B9 antibody only in permeabilized T24 cells. N-cadherin was detected by the GC-4 antibody in both non-permeabilized and permeabilized cells. Thus, only T24 cells expressed N-cadherin protein and only GC-4 clone recognized the extracellular domain of this adhesion molecule. Therefore, we used GC-4 clone for AFM exploration of the cells.

**Fig. 3:**
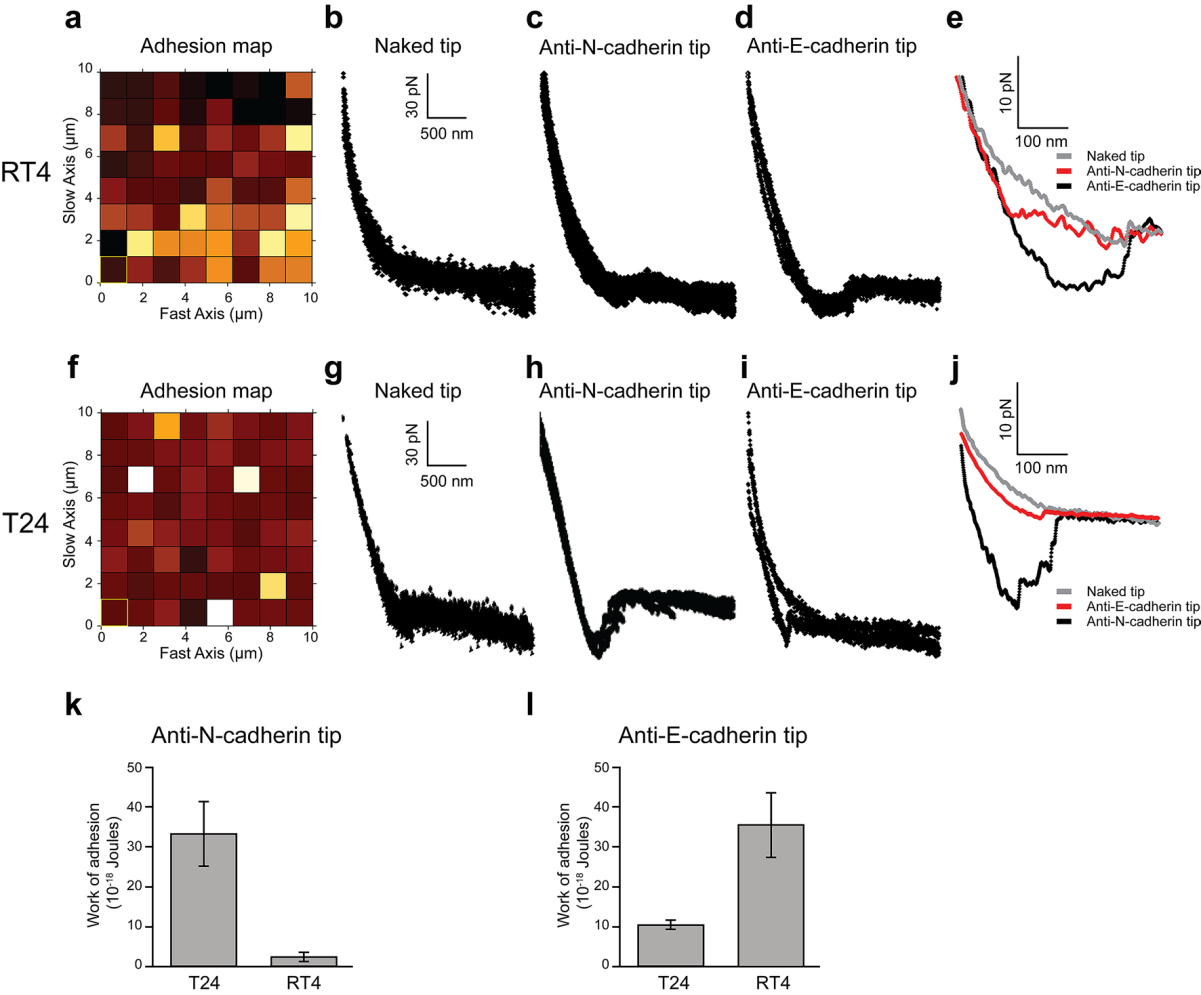
Mapping of N- and E-cadherins on RT4 and T24 cells. **a** Adhesion map and **b**–**e** representative force curves obtained on RT4 cells with a naked tip (**b**), an anti-N-cadherin functionalized AFM tip (**c**), and an anti-E-cadherin-functionalized AFM tip (**d**). **e** Overlap of selected representative force curves from **b**–**d**. **f** Adhesion map and **g**–**j** representative force curves obtained on T24 cells with a naked tip (**g**), an anti-N-cadherin-functionalized AFM tip (**h**), an anti-E-cadherin-functionalized AFM tip (**i**). **j** Overlap of selected representative force curves from **g**–**i**. **k** Quantification of the interaction measured between the anti-N-cadherin-functionalized AFM tip and T24 or RT4 cells. The work of adhesion (expressed in Joule) was measured in the two types of cells. Interaction was significantly different between the two types of cells. **l** Quantification of the interaction measured between the anti-E-cadherin-functionalized AFM tip and T24 or RT4 cells. The work of adhesion (expressed in Joule) was measured in the two types of cells. Interaction was significantly different between the two types of cells. More than 30 curves were registered in each condition. *z* scale of adhesion map was 90 nN, and pulling speed of all curves was 2 µm/s

In order to help in characterization of cadherin expressed by RT4 and T24 cells, we engaged force spectroscopy with AFM tip functionalized with anti-E or N-cadherin antibodies. The experiments were carried out in liquid medium on non-permeabilized living cells and that is why we used the clone GC-4 for N-cadherin antibody. According to the previously described results, RT4 cells were used as a negative control since they did not express N-cadherin and as a positive control for E-cadherin expression. We realized adhesion maps on cells, at 8*8 and 16*16 pixels, on area of 50–100 µm^2^, as shown in Fig. 3a for RT4 cells and in Fig. 3f for T24 cells. RT4 and T24 cells were mapped using an anti-E- or anti-N-cadherin antibody grafted on the AFM tip. Our results showed the presence of E-cadherin at the surface of RT4 cells using a tip functionalized with anti-E-cadherin antibody (Fig. 3d) with curves presenting an interaction peak, in comparison with a tip functionalized with an anti-N-cadherin antibody (Fig. 3c) or a naked tip (Fig. 3b). Selected representative force curves registered on RT4 cells in the three previously mentioned conditions are overlayed in Fig. 3e (grey curve: naked tip; red curve: a tip functionalized with an anti-N-cadherin antibody; black curve: a tip functionalized with an anti-E-cadherin antibody). Our study revealed the presence of N-cadherin on T24 cells through a high frequency of force curves presenting a reproducible pattern with a recurrent force peak area (Fig. 3h). These tip/cells interactions were specific of N-cadherin, since neither a naked AFM tip (Fig. 3g) nor a tip functionalized with an E-cadherin antibody (Fig. 3i) induced such interaction peaks on T24 cells. Selected representative force curves registered on T24 cells in the three previously mentioned conditions are overlayed in Fig. 3j (grey curve: naked tip; black curve: a tip functionalized with an anti-N-cadherin antibody; red curve: a tip functionalized with an anti-E-cadherin antibody). The peaks of interactions were represented in the form of histograms (Fig. 3k, l). Interactions with anti-N-cadherin antibody were significantly higher for T24 than for RT4 cells (Fig. 3k), while interactions with anti-E-cadherin antibody were significantly higher for RT4 than for T24 cells (Fig. 3l). Interactions with anti-N-cadherin antibody in RT4 cells and those with anti-E-cadherin antibody in T24 cells were aspecific since RT4 cells did not express N-cadherin and T24 cells did not express E-cadherin (see immunoblotting experiments in Fig. 2a). These interactions corresponded to the background. Thus, the AFM analysis has allowed to determine the density of cadherins on the surface of RT4 and T24 cells through the measurements of the interaction forces involving these adhesion molecules. N-cadherin was specifically detected on T24 cells, while E-cadherin was specifically detected on RT4 cells. We can claim for a specific recognition signal, since the same location was repetitively scanned, and the adhesion map (data not shown) displayed that the tip-surface interactions were stable and almost located at the same sites on subsequent scans of the same area.

### GW501516 decreases N-cadherin level in T24 cells

**Fig. 4:**
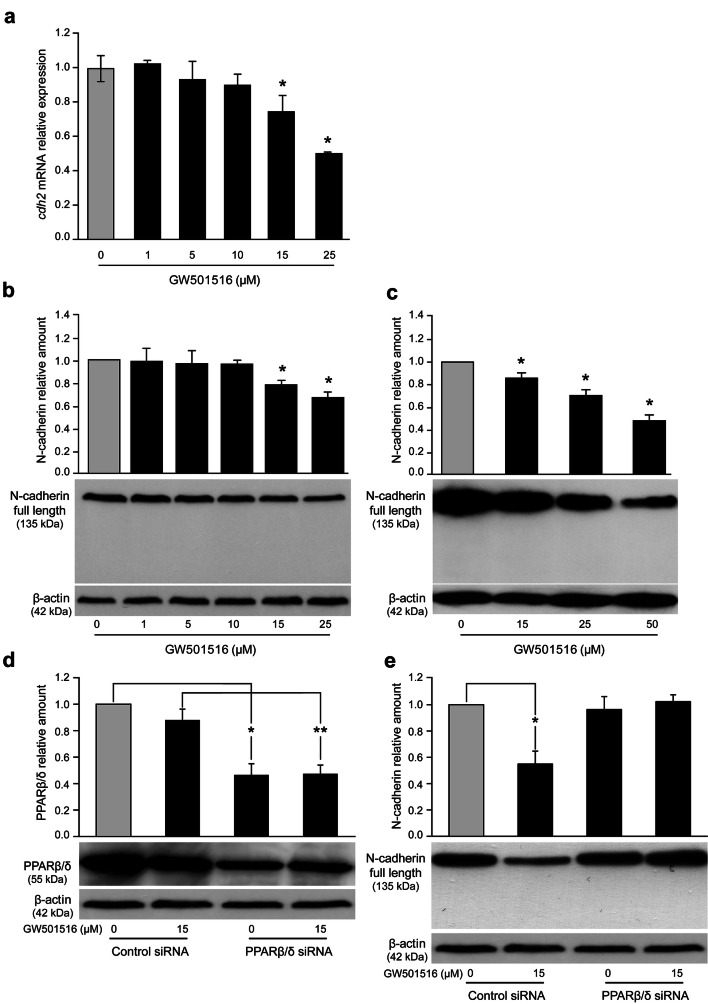
GW501516 impact on N-cadherin level in T24 cells. Cells were exposed for 24 h to vehicle or PPARβ/δ activator at the indicated concentrations. **a** RTqPCR analysis of *cdh2* mRNA expression. Fold inductions represent comparison with control cells (set at 1). **b**, **c** N-cadherin detection was determined by Western blotting analysis from total protein extracts with GC-4 antibody. β-actin was used as an internal loading control. The density of each band in immunoblots was measured by using ImageJ and N-cadherin levels were determined as arbitrary units in control and treated cells. The amount of N-cadherin protein was normalized to that of β-actin. Data are expressed as means ± SEM of three independent experiments performed in triplicate. *P < 0.05 vs control cells. **d** Immunoblotting detection of PPARβ/δ after siRNA-mediated knockdown of PPARβ/δ expression in cells stimulated or not with 15 µM GW501516. β-actin was used as an internal loading control. The density of each band in immunoblots was measured by using ImageJ and the amount of PPARβ/δ was normalized to that of β-actin. Data are expressed as means ± SEM of two independent experiments performed in triplicate. *P < 0.05 vs control of the control siRNA-transfected cells; **P < 0.05 vs the GW501516 of the control siRNA-transfected cells. **e** PPARβ/δ-dependent effect of GW501516 on N-cadherin decrease. T24 cells were transfected by control siRNA or PPARβ/δ siRNA at a concentration of 50 nM. N-cadherin detection was determined by Western blotting analysis from total protein extracts. β-actin was used as an internal loading control. The density of each band in immunoblots was measured by using ImageJ; N-cadherin levels were determined as arbitrary units in control and treated cells. The amount of N-cadherin was normalized to that of β-actin. Data are expressed as means ± SEM of two independent experiments performed in triplicate. *P < 0.05 vs control of the control siRNA-transfected cells

**Fig. 5:**
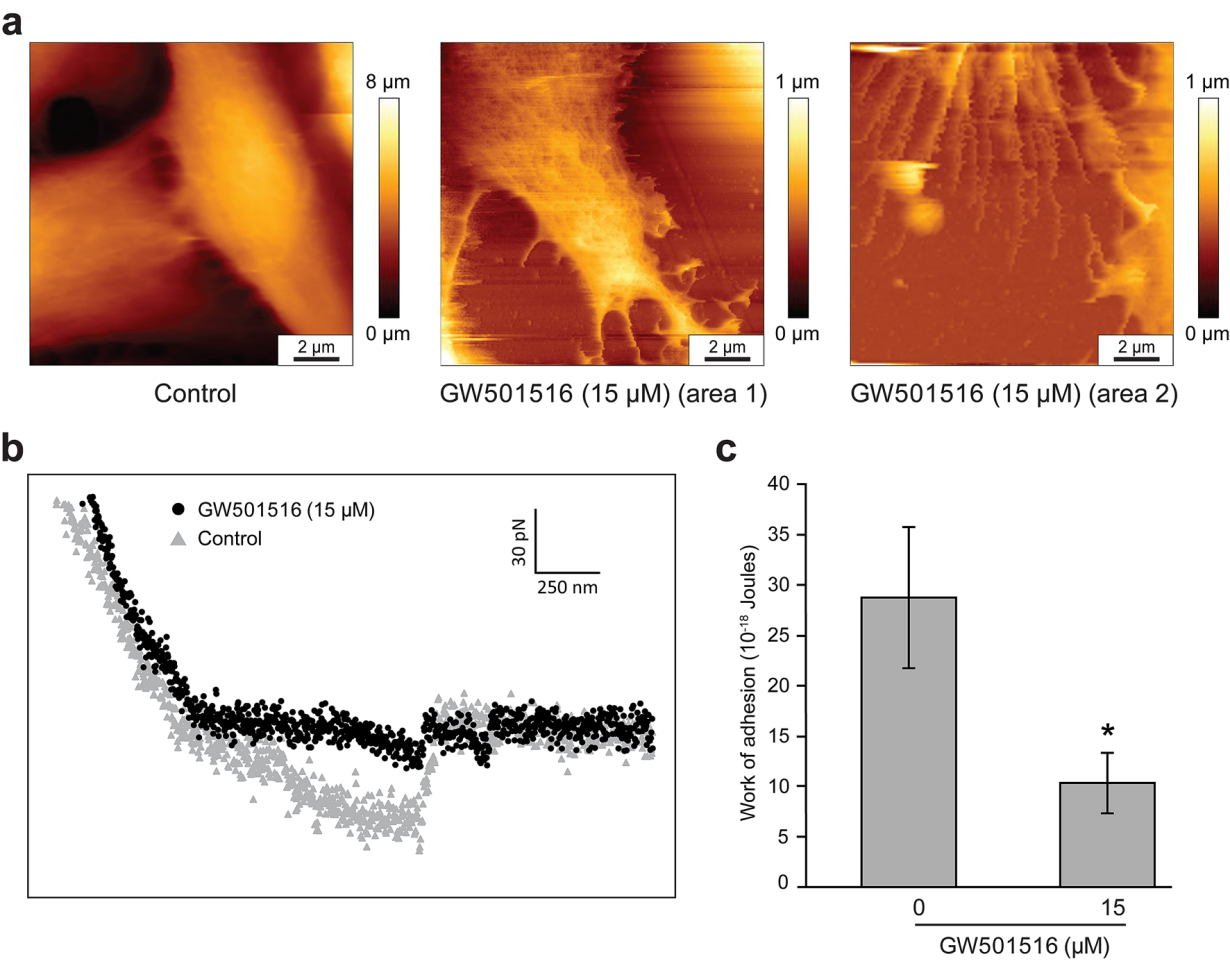
AFM analysis of GW501516 impact on T24 cells. **a** AFM images of control T24 cells (left) and of two different cells (middle and right) after a 15 µM GW501516 incubation. **b** Typical force curves registered with an anti-N-cadherin-functionalized AFM tip on control T24 cells (in grey) and after a 15 µM GW501516 incubation (in black). **c** Quantification of the interaction measured between the anti-N-cadherin-functionalized AFM tip and T24 cells without and after a 15 µM GW501516 treatment. The work of adhesion (expressed in Joule) was measured in the two conditions, and more than 30 curves were registered in each. Interaction was significantly different between the two conditions. Pulling speed of all curves was 2 µm/s

To investigate the effect of GW501516 on N-cadherin level, T24 cells were treated with increasing concentrations of the PPARβ/δ activator for 24 h and assessed for mRNA and protein expression by RTqPCR and Western blotting analyses, respectively. As shown in Fig. 4a, *cdh2* mRNA level was significantly decreased at 15 and 25 µM GW501516. At the protein level, N-cadherin decreased at the same concentrations up to 50 µM GW501516 (Fig. 4b, c). In the following experiments, we used the concentration of 15 µM since from this dose a significant diminution of N-cadherin was observed. To determine whether PPARβ/δ transactivation was required for GW501516-mediated decrease of N-cadherin, RNA interference strategy to knockdown PPARβ/δ protein was applied (Fig. 4d). N-cadherin downregulation by GW501516 was suppressed in the presence of PPARβ/δ siRNA (Fig. 4e). These data demonstrated that the PPARβ/δ agonist decreased N-cadherin expression through a PPARβ/δ-dependent molecular mechanism. Using AFM and compared to control cells, T24 cells presented also a particular morphology from this concentration of 15 µM PPARβ/δ activator. Especially, their contour was largely weakened and even destroyed (Fig. 5a, area 1). As noted in Table 1, the length of the cells had slightly decreased. On the other hand, the width decreased from 16.5 ± 1.5 to 8.2 ± 3.1 µm. In addition, the average roughness (Ra) decreased from 273 ± 84 to 124 ± 16 nm suggesting a modification of the cell surface ultrastructure. A high magnification of the same image showed that these cell impairments appeared through an abundance of thin and fragile membrane expansions surrounding cells (Fig. 5a, area 2). Moreover, force spectroscopy engaged on T24 cells incubated with GW501516 at 15 µM for 24 h revealed a significant decrease of 1/the appearance frequency of force curves presenting peaks, in comparison with untreated cells (Fig. 5b) and 2/the peak area for the rare force curves that presented interaction peaks when all values were plotted as an histogram (Fig. 5c). Therefore, GW501516 decreased N-cadherin from 15 µM and this diminution was detected with more sensitivity by AFM.

### A high non-toxic dose of GW501516 blocks T24 cell wound closure

**Fig. 6:**
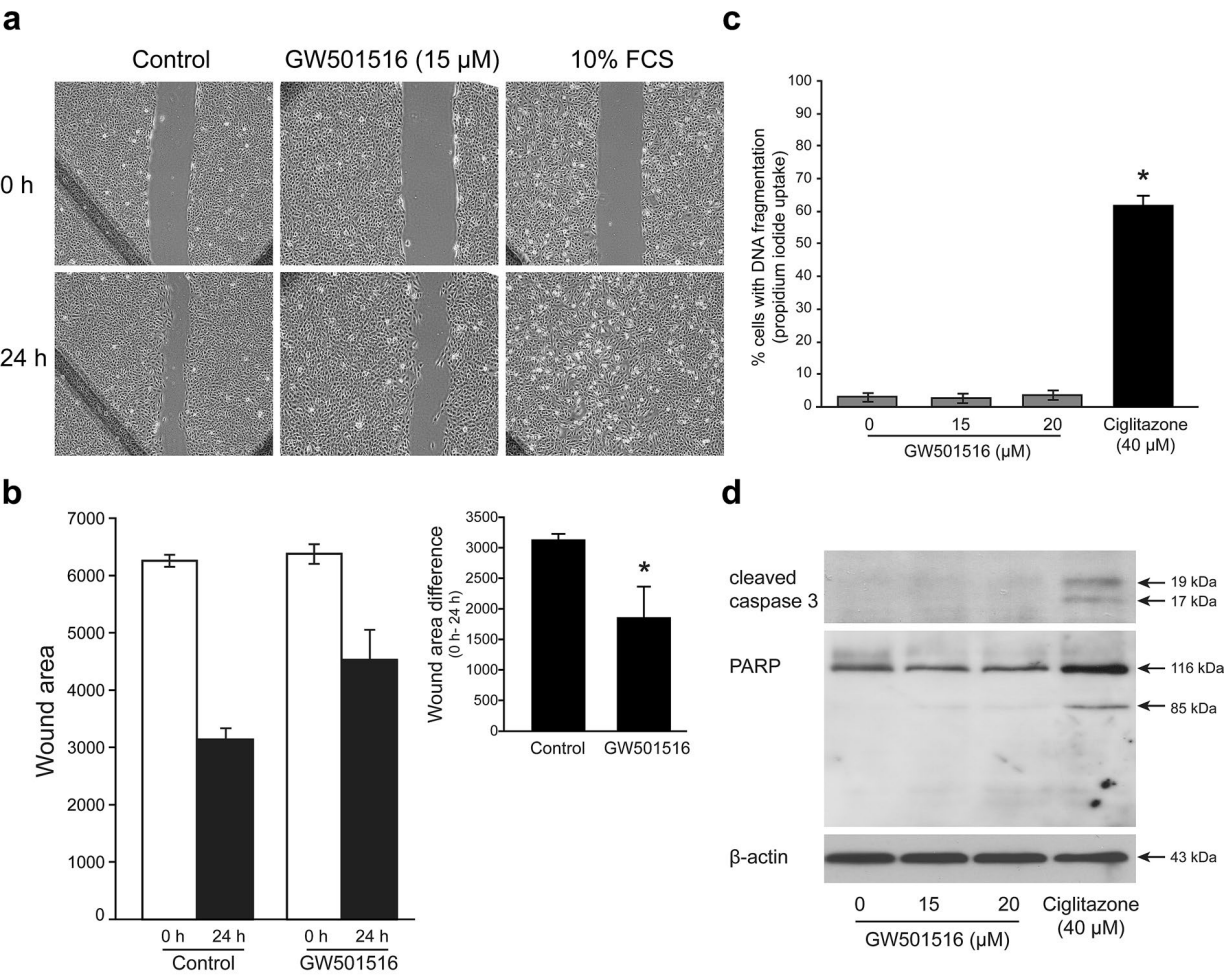
GW501516 effect on T24 cell wound closure. **a** Representative microscopy images of the scratch-wound healing assay captured at 0 h and 24 h. Scratched T24 confluent monolayers were incubated with or without 15 µM GW501516 or 10% FCS (positive control) for 24 h after injury (× 10 magnification). **b** Wound closure measurement by the calculation of the wound area at 0 h and 24 h in treated and untreated cells; *Insert*, graph representing the wound area difference between 0 and 24 h for both control cells and GW501516-treated cells. Data are expressed as the mean ± SEM of two independent experiments in duplicate. *P < 0.05 vs control cells. **c** Cells were treated with 15 or 20 µM GW501516 or 40 µM ciglitazone (PPARγ activator used as a positive control of cell death) for 24 h. The percentage of cells showing the hypodiploid DNA content (sub-G1 peak) was evaluated by flow cytometry analysis after propidium iodide staining. Data are expressed as the mean ± SEM of three independent experiments performed in triplicate. *P < 0.05 vs control cells. **d** Total T24 cell protein extracts were subjected to a Western blotting analysis for the study of caspase 3 and PARP cleavage. β-actin was used as an internal loading control

To determine whether GW501516 treatment affected cell migration, we evaluated wound closure after a 24 h-exposure of T24 cells upon 15 µM drug. We chose this concentration because from this dose we observed a significant decrease of N-cadherin expression (Fig. 4a–c) after RTqPCR and immunoblotting analyses, a modification of T24 cell morphology (Fig. 5a) and a decrease of the appearance frequency of force curves presenting interaction peaks (Fig. 5b) after AFM observation. Used as a positive control, an incubation with 10% FCS led to a complete wound filling after 24 h. On the other hand, the PPARβ/δ activator blocked the wound closure into the wounded area compared with that in the control cells (Fig. 6a). Thus, GW501516 at 15 µM significantly decreased the wound closure rate by 40% (Fig. 6b). We checked that the inhibition of the wound closure by GW501516 was not due to cell death. After a 24 h-treatment with 15 or 20 µM GW501516, cells were assessed for propidium iodide stained DNA content using flow cytometric analysis. There was no significant increase of the sub-G1 population compared to untreated cells and unlike what was observed in the presence of 40 µM ciglitazone (60% cells with fragmented DNA) (Fig. 6c). In addition, cleaved caspase 3 was not detected and consequently the downstream target PARP (Poly(ADP-Ribose) Polymerase) was unprocessed (Fig. 6d) upon GW501516 exposure unlike the positive control of cell death when cells were incubated in the presence of ciglitazone. These overall data showed that these concentrations of GW501516 were not cytotoxic on T24 confluent cells since they did not induce apoptotic cell death, but were probably able to inhibit cell migration.

## Discussion

The aberrant expression of N-cadherin in carcinomas is well documented from many experimental models. It is associated with the acceleration of cancer cell migration and invasion and, therefore, tumor aggressiveness promoting metastasis development. In bladder cancer, in particular, we demonstrated that non-muscle-invasive tumors confined to the lamina propria and expressing N-cadherin will progress to a muscle-invasive disease [6]. Moreover, its expression appeared in 40% of patients with muscle-invasive urothelial carcinomas and this was associated with a poor recurrence-free survival [26].

The *cis* adhesion between N-cadherin at the plasma membrane of a cell involves a lateral clustering through the EC1 domain of one monomer and the EC2 domain of another one. Then, N-cadherin dimers interact through the EC1 motifs between neighboring cells [27]. This is essential for stable and mature adhesion junctions. Given the role of this adhesion molecule in cancer, the utility to antagonize its functions has been investigated. Different strategies have been developed including monoclonal antibodies (GC-4, 1H7, 2A9) and peptides (ADH-1) that have displayed anti-tumor efficacy in vitro and in vivo. Thus, the GC-4 monoclonal antibody directed against the EC1 domain of N-cadherin extracellular part inhibited both N-cadherin-mediated adhesion and T24 bladder cancer cell invasion through Akt signaling inactivation [28]. Furthermore, acute myeloid leukemia cells pre-treated with GC-4 blocked bone marrow homing of circulating cancer cells [29]. The stable cyclic peptide ADH-1 containing the HAV (His-Ala-Val) motif is able to compete with the HAV sequence localized in the EC1 domain of N-cadherin disrupting N-cadherin-dependent functions through inhibition of lateral assembling of N-cadherin monomers [30]. Among N-cadherin antagonists, only ADH-1 has been tested in clinical trials. In combination with melphalan, it seemed promising to treat melanoma [31].

For the first time, we demonstrated that GW501516 decreased N-cadherin full length at the mRNA and protein level. And this effect was strongly diminished when the level of PPARβ/δ receptor was decreased by specific siRNA. So, the action of the ligand was dependent on the presence of the nuclear receptor. To date, no PPRE sequence has been identified on the promoter of the *cdh2* gene encoding for N-cadherin. The *cdh2* gene encoding for N-cadherin is regulated by several transcriptional factors either negatively (p63 [32], CREB (cAMP Response Element-Binding Protein) [33]) or positively (Twist [34], SMAD3/4 [35], AP-1 (Activator Protein-1) [36], ERRα (Estrogen-Related Receptor alpha) [37], NFκB (Nuclear Factor kappa B) [38]). In our case, GW501516 could decrease an activator or increase a repressor of *cdh2* transcription. According to the published data, PPARβ/δ agonists such as L-165041 and GW501516 inhibited the expression and activity of MMP-9 induced by TGFα (Transforming Growth factor α) in HaCaT keratinocytes. These preventing effects were mediated through the reduction of c-FOS binding to an AP-1 binding site [39]. Other studies reported the inhibitory effect of GW501516-activated PPARβ/δ on the NFκB signaling. Unliganded PPARβ/δ is known to be associated with the transcriptional repressor BCL6 (B-cell lymphoma 6), a mechanism responsible for the anti-inflammatory properties of this receptor [40] and BCL6 is an inhibitor of NFκB activity [41]. As demonstrated in pancreatic cancer cells upon binding of GW501516, PPARβ/δ decreased TNFα (Tumor Necrosis Factor α)-induced NFκB activity leading to the induction of anti-inflammatory genes and consequently to the inhibition of pro-inflammatory genes through the dissociation of BCL6 [42]. Further investigations are needed to clarify the negative regulation of *cdh2* mediated by GW501516 in T24 cells.

The decrease in the rate of *cdh2* mRNA was correlated with the reduction of N-cadherin protein level and this was associated with a decrease in the amount of N-cadherin present at the cell surface as evidenced by the AFM experimental data. Indeed, in the present study, we described for the first time the impact of the GW501516 molecule on both confluent T24 cell morphology and N-cadherin expression. Our results showed that the cell morphology was impaired by the drug. While control T24 cells were nicely spread on the substrate with large and homogeneous membrane expansions, GW501516-treated T24 cells seemed to be fragilized and to lose adhesion on the substrate. Indeed, treated cell surrounding areas were like unraveled and showed really thin membrane extensions. Control T24 cells presented a roughness average (Ra) in the range of 250 nm. The value of this parameter was higher than that already published (80 nm) [43] probably due to the fact that we qualified living and not fixed cells. The decrease of both the width and the roughness after treatment could be in favor of the detachment of cells from the substrate and a decrease of membrane proteins at the cell surface. This observation could be related to the fact that the drug impacted the expression of N-cadherin. By the way, a GW501516-mediated decrease of N-cadherin expression was observed that was confirmed by force spectroscopy experiments. Indeed, lower frequency and intensity of interaction peaks were registered when treated T24 cells were scanned with the anti-N-cadherin tip that was correlated with a decrease in N-cadherin expression emphasized by SDS-PAGE. Our nanoscale approach with AFM offers, in addition to a biochemical investigation, a sensitive single cell characterization method to study and quantify the effect of a drug on cell topography, morphology and membrane protein expression. It's worth noting that AFM analysis detected a major effect of GW501516 at the dose of 15 µM on N-cadherin expression in T24 cells whereas at this concentration the impact of the drug was observed with a smaller scale by RTqPCR and Western blotting techniques.

The role of PPARβ/δ in carcinogenesis is widely debated since this nuclear receptor has pro- or anti-tumor properties. The present findings showed that GW501516 suppressed invasive bladder cancer cell wound filling. Other studies reported also that this drug inhibited both migration and invasion of breast cancer cells through the downregulation of thrombospondin-1 and the upregulation of its degrading protease ADAMTS1 (a disintegrin and metalloprotease with thrombospondin-1 motifs) [14]. In addition, GW501516-mediated activation of PPARβ/δ led to the reduced expression of MMP-9 after the BCL6 transcriptionnal repressor dissociation and consequently to the decrease of pancreatic cancer cell invasion capacities [13]. In contrast to our results, other experimental works reported that the activation of PPARβ/δ by GW501516 significantly increased the migration and invasion of highly metastatic melanoma cells through the upregulation of Snail expression (a transcriptional repressor of *cdh1* gene encoding for the E-cadherin) and the decrease of E-cadherin expression [44], enhanced human cholangiocarcinoma cell proliferation [45], or enhanced colorectal cancer in APC mutated mice [46].

Our study brings interesting first results that need to be deepened. Thus, we planned new experiments that are in progress to elucidate the molecular mechanisms involved in GW501516-mediated decrease of N-cadherin and to demonstrate that this reduction of N-cadherin could be responsible for the decrease of T24 cell migration. Interestingly, this drug or possibly other PPARβ agonists might provide clinical benefit by preventing bladder cancer cell spreading through the N-cadherin decrease.
